# HDAC8 inhibitor attenuates airway responses to antigen stimulus through synchronously suppressing galectin-3 expression and reducing macrophage-2 polarization

**DOI:** 10.1186/s12931-020-1322-5

**Published:** 2020-02-28

**Authors:** Meng-lu Li, Xin-ming Su, Yuan Ren, Xuan Zhao, Ling-fei Kong, Jian Kang

**Affiliations:** Department of Respiratory and Critical Care Medicine, Institute of Respiratory Diseases, The First Affiliated Hospital of China Medical University, Shenyang, 110001 Liaoning China

**Keywords:** Histone deacetylases8, Galectin-3, PCI-34051, M2 macrophage polarization

## Abstract

**Background:**

This study was to investigate of the mechanism by which histone deacetylase (HDAC) 8 inhibitor ameliorated airway hyperresponsiveness (AHR) and allergic airway inflammation.

**Methods:**

Mice were sensitized and then treated with budesonide (BUD) or PCI-34051 (PCI) prior to exposing to normal saline (NS) or ovalbumin (OVA). The raw264.7 cells were treated with interleukin (IL)-4 and PCI or shRNA alone. Repetitive measurements of enhanced pause (Penh) were executed by increasing concentrations of acetyl-β-methacholine chloride (0 - 50 mg/ml). Cells in bronchoalveolar lavage fluid (BALF) and pathological changes of lungs were examined, respectively. The expression levels of HDAC8, Galecitn (Gal)-3, CD68, CD86, CD163, Arg1 and NOS2 in lungs were measured. Co-regulation of HDAC8 and Gal-3 proteins was observed by immunofluorescence staining and co-immunoprecipitation assay (Co-IP).

**Results:**

Significant increases in Penh and IL-4 level were detected with a large inflammatory infiltrate, comprised predominantly of macrophages and eosinophils, into the BALF in OVA-exposed lungs. HDAC8, Gal-3, CD68, CD86, CD163, Arg1 and NOS2 proteins were over-expressed with the significant changes in the Arg1 and NOS2 mRNA levels in the lungs and the IL-4-treated cells. PCI intervention obviously reduced the counts of CD163^+^ cells. Furthermore, Gal-3 knockdown suppressed Arg1 expression in the cells. Immunofluorescence staining displayed simultaneous changes in HDAC8 and Gal-3 expression in the investigated samples. Treatment with PCI resulted in synchronous reduction of HDAC8 and Gal-3 expression in the Co-IP complexes.

**Conclusions:**

The HDAC8 inhibitor ameliorates AHR and airway inflammation in animal model of allergic asthma through reducing HDAC8-Gal-3 interaction and M2 macrophage polarization.

## Background

Histone deacetylases (HDACs) include four classes of enzymes that catalyze the removal of acetyl functional groups from the lysine residues of both histone and nonhistone proteins [1]. HDAC8 is one of the class I HDACs and has been encoded by its gene in humans [2, 3]. Since HDAC8 has been overexpressed in a variety of human cancers, it has been employed as one of the attractive therapeutic anticancer targets based on structural features of this protein and inhibitory activity and selectivity of targeting drugs [4–6]. Additionally, an increase in the enzyme activity is linked to a number of lung diseases such as asthma and COPD [7, 8], HDAC8 inhibitors are applied for treatment of asthma through decreasing infiltration of inflammatory cells and content of cytokines in lungs [7, 9]. It has been reported that increased influx of macrophages in lungs has been recognized as the pathogenesis of allergic asthma [10], whereas the macrophages are polarized into two phenotypes of M1 (classically activated macrophages) and M2 (alternatively activated macrophages) in inflammatory responses to pathogens [11, 12]. M1 and M2 macrophages are distinguished by the differential expression of molecules such as nitric oxide and arginase [13]. However, HDAC8-related macrophage polarization in asthma is still largely unknown. Gal-3 as a member of the beta-galactoside-binding protein family plays an important role in cell-cell adhesion, cell-matrix interactions and inflammation [14]. This protein also involves the pathogenesis of asthma [15].

PCI-34051 is a potent and specific HDAC8 inhibitor with > 200-fold selectivity over other class I HDACs [16]. Given many studies in which the inhibitor displays a therapeutic benefit in diseased conditions [17–19], it is necessary to decipher the role of the PCI-targeted enzyme in allergic lung inflammation and to gain the understanding of the relatively extensive events associated with its inhibition at cellular and molecular levels. This would be important to broaden a potential therapeutic window in identifying and developing novel inhibitors for the treatment of asthma.

This study was to investigate the effects of HDAC8 inhibitor PCI-34051 on OVA-exposed lungs and IL-4 treated macrophages. Our results indicate that there is interaction between HDAC8 and Gal-3 proteins, which influences macrophage-2 polarization in the events of AHR and allergic airway inflammation.

## Methods

### Preparation of animals

Specific pathogen-free, female BALB/c mice (6 wks) weighting about 20 g were purchased from the Center for Experimental Animals at China Medical University (Shenyang, P. R China). Those animals were housed at animal facility of our hospital for the duration of the experiments. All procedures were reviewed and approved by the Institutional Animal Care and Use Committee of The First Affiliated Hospital of China Medical University.

Forty-eight mice were randomly divided into four groups of twelve animals for each. The protocol for making animal model is modified according to previous studies [20, 21]. Mice were sensitized with intraperitoneal injection of an equivalent volume of 0.9% saline (NS) or ovalbumin (OVA, 20 μg in saline) complexed with 2 mg Al (OH)_3_ in 0.2 ml NS on days 0, 7 and 14. On day 21, mice started to receive aerosol inhalation of NS or OVA (2 mg/ml) at a flow rate of 3 ml/min for 30 min by an ultrasonic nebulizer (Jiangsu Yuyue Medical Equipment & Supply Co. Ltd., China). This procedure was carried out once a day for seven consecutive days. Some of mice inhaled budesonide (BUD, 2 mg) for 30 min or received IP injection of PCI-34051 (PCI, 0.5 mg/kg) once a day prior to the challenge. The animals in the control were only exposed to NS for the same time period. Measurements in this study were performed 24 h after the final aerosol.

The left lungs of three mice in each group were fixed with 4% paraformaldehyde for hematoxylin and eosin (H&E) and Periodic Acid-Schiff (PAS) staining. The right lungs of the same mice were fixed by vascular perfusion with formaldehyde fixative solution (85 mM Na_2_HPO_4_, 75 mM KH_2_P0_4_, 4% paraformaldehyde, and 14% (v/v) saturated picric acid, pH 6.9). After that, the lungs were embedded in paraffin and frozen at − 80 °C for the assay of immunocytochemistry (IHC) and immunofluorescence (IF). The left and right lungs from another three mice in each group were harvested and the frozen samples were stored at − 80 °C for protein and mRNA analysis.

### Measurement of airway responsiveness

Six mice from each group were used to evaluate pulmonary resistance in vitro. Airway responses to inhaled acetyl-β-methacholine (MCh) were measured non-invasively in conscious, unrestrained mice using barometric whole-body plethysmography (EMKA Technologies, Paris, France). Airway responsiveness was expressed in enhanced pause (Penh), which is a measure of bronchoconstriction [22]. Briefly, mice were placed in a whole-body chamber, and basal readings for airway responsiveness were obtained and then averaged for 3 min. Subsequently, the animals were aerosolized with increasing concentrations of MCh (0–50 mg/ml). The readings for the Penh value were taken after each nebulization. The degree of airway resistance was expressed as an increase in the value relative to the baseline.

### Cell counts in bronchoalveolar lavage fluid and serum IL-4 level

Lung after measuring Penh was lavaged by instillation and withdrawal of 1.0 ml of NS (× 3 times) through a tracheal cannula, and an equal volume of BALF was collected from each mouse. The BALF sample was centrifuged (1200 rpm × 5 min) at 4 °C and total cells were counted using a hemocytometer under a microscope. Two hundred microliter of a cell suspension from each sample was applied to a glass slide using a cytospin (1000 rpm × 10 min) and then the slide was stained with Wright-Giemsa for the differential cell counts.

The serum was obtained after blood clot is removed by centrifuging (2500 rmp × 15 min) for ELISA. Antibody for the murine IL-4 was purchased from Pharmingen (R&D system Inc. NE USA) and the IL-4 level was determined using Infinite® 200 PRO (Tecan Trading AG, Switzerland) according to the manufacturer’s direction.

### Histopathologic examination

Lungs were surgically removed and were inflated in 4% paraformaldehyde for 24 h. Lungs of mice were embedded in paraffin and cut into a 4-μm section which was stained with H&E or PAS solution for evaluating inflammatory conditions and the presence of mucus production in the lungs. The stained specimens were visually observed under a light microscope (Olympus, Japan) at a magnification of 40 and photographed to compare morphological changes.

### Cell culture

The RAW264.7 murine macrophage-like cell line was used for investigation of mechanism of action of PCI based on the fact that alveolar macrophages (AM) can polarize into many different phenotypes in allergic asthma [10]. The cells purchased from The Cell Bank of Type Culture Collection of Chinese Academy of Sciences (Shanghai P.R. China) and cultured in Dulbecco’s Modified Eagle’sMedium (DMEM) supplemented with 10% fetal bovine serum (FBS) and 1% penicillin/streptomycin sulfate. Cells were grown in 6-well plates to 90% confluence and then stimulated with 20 ng/ml recombinant mouse IL-4 (R&D Systems, USA) for 24 h since the cytokine induces macrophage activation [23, 24]. Some cells were treated with HDAC8 inhibitor (PCI, 10 mM) for 30 min prior to stimulation with IL-4 or NS as a control.

### Immunohistochemical and immunofluorescent staining

To provide visual details about macrophage phenotypes and protein abundance in lung tissues and the cells, IHC and IF procedures were used according to the manufacturer’s protocol. Briefly, the slices of lung samples were mounted on slides and dehydrated using alcohol washes. The sections were incubated with primary antibodies of CD68 at 1:100 (sc-20,060; Santa-Cruz Biotechnology, CA, US), CD86 at 0.5 μg/ml (NBP2–25208; Novus Biologicals, CO, US) and CD163 at 1:500 (ab182422; Abcam, Cambridge, UK) overnight at 4 °C. On the following day the sections were incubated with a HRP-conjugated secondary antibody at 1:5000 (Beijing Zhongshan Jinqiao Biotechnology Co., Ltd., Beijing, China) for 1 h at 37 °C. Reaction with a 3,3′-Diaminobenzidine (DAB) on the sections can produce a brown product at the site of the target antigen. Slides were washed three times with phosphate-buffered saline (PBS) and observed using a light microscope (Olympus BX51, Japan) at × 400 magnification.

To investigate co-distribution of HDAC8 and Cal-3 in the tissues and the cells, we used rabbit and rat primary antibodies at 1:100 (sc-11,405 and sc-23,938; Santa-Cruz Biotechnology, CA, US) to detect two distinct antigens on the same samples. Double staining IF was processed with simultaneous incubation of two sets of the antibodies (Santa Cruz Biotechnology, Dallas, TX). Briefly, the sample section was washed twice in TBST (Tris 0.05 M pH 7.5; 0.01% Tween 20) after fixing with acetone and then blocked with 1% BSA for 30 min at a room temperature (RT). Afterwards, the section placed in a humidified box was co-incubated with the mixture of two primary antibodies in PBS containing 1% BSA) and stayed overnight at 4^0^ C. After washing three times with TBST, the section was incubated with the mixtures of a goat-anti-rabbit and a goat-anti-rat IgG (H&L) secondary antibodies at 1:400 (Cell Signaling Technology, Inc., MA, US) at RT for 1 h in dark. Color responses were raised in different species with two different fluorochromes (TRITC-conjugated against rabbit and FITC-conjugated against rat). The section was stained with DAPI for 10 min and was mounted on a glass slide, and coverslipped with anti-fading medium. Areas of interest on the stained tissue were visualized using a confocal microscope and photographed using a Digital Sight DS-Fi1 camera (Nikon, Melville, NY) and the NIS-Elements software package (Nikon).

### Electrophoresis and quantification of proteins

Lung tissues were collected and the lysate was prepared using a rapidly oscillating masher in modified RIPA buffer (50 mM Tris-HCl, pH 7.4, 1% Triton X-100, 0.2% sodium deoxycholate, 0.2% sodium dodecylsulfate, 1 mM sodium ethylenediaminetetraacetate, 1 mM phenylmethylsulfonyl flouride, 5 μg/ml of aprotinin, 5 μg/ml of leupeptin). Macrophages were lysed in the buffer supplemented with protease inhibitors and homogenized. Supernatants from lungs and cells were obtained with centrifuge (12,000 x g) for 20 min at 4 °C, respectively. Protein concentrations of the supernatants were determined by the BCA method (Pierce Biotechnology, Rockford, IL). The supernatants were stored at − 70 °C until use. Briefly, aliquots of lung tissue and cell lysates (40 μg/well and 10 μg/well) were loaded onto a 10% SDS polyacrylamide gel. The electrophoresed proteins were transferred to nitrocellulose membrane and blocked with 5% nonfat dried milk in Tris-buffered saline (TBS) at 4 °C for 120 min. The membrane was individually incubated with primary antibodies (1:1000) to HDAC8 (sc-11,405), Gal-3 (sc-23,938), Arginase-1 (Arg-1, 93,668, D4E3M™) and inducible nitric oxide synthase2 (NOS2, abcam 15,323) overnight at 4 °C and then washed with TBST (TBS, 0.1% Tween-20). The membrane was incubated with peroxidase-conjugated secondary antibody (1:5000) at RT for 60 min and washed with TBST again. A mixture of western blotting detection reagent I and II (GE Healthcare Life Sciences, Piscataway, NJ) was poured on the membrane at RT. The bands for both proteins on the membrane were visualized by autoradiogaphy. To compare target protein abundance in samples, proteins were quantified using Image-J and the intensity of each protein band was expressed as a value divided by the intensity of the GAPDH protein band.

### Co-immunoprecipitation of protein complexes

A protein complex was isolated using Dynabeads® Co-Immunoprecipitation Kit (26,149, Thermo Scientific Pierce) according to vendor’s instructions. Briefly, the cell lysate was transferred to chilled fresh tube after centrifuging at 12,000 x g for 30 min at 4 °C and protein concentration was determined in the supernatant. A predetermined amount of antibody (HDAC8 and IgG) was added to each sample tube and the mixture was kept overnight at 4 °C on a rotary mixer, 40 μl of protein A/G Sepharose® beads slurry was added to each tube and incubated for 1 h at 4 °C. Protein A/G Sepharose® beads were collected by centrifugation (2000 g × 2 min) at 4 °C after washing with PBS. The beads with the antigen-antibody complex were analyzed by western blotting with Gal-3 and HDAC8 antibodies. Input (cell lysates) and an isotype IgG were used as negative controls to load same amount of the protein as the sample.

### shRNA transfection

Gal-3 shRNA and non-targeting shRNA (NTC) were purchased from Shanghai GenePharma Co., Ltd. (Shanghai, P.R. China). Briefly, macrophages were incubated with RNase free H_2_O, shRNA1, shRNA2 and NTC. Transfection of shRNA was carried out with Lipofectamine 2000 (Invitrogen, Carlsbad, CA, USA) according to the procedure recommended by the manufacturer. The cells were harvested 24 h post-transfection. The level (%) of the silencing gene was expressed as a percentage in reference to the expression level of NTC.

### qPCR analysis

Arg1 and NOS2 mRNA expressions were assessed by using SYBR Premix Ex TaqTM (Takara, Japan) and the mRNA levels were normalized to GAPDH housekeeping gene. The following sense and antisense primer sequences were used: Arg1, 5′- AGCTCTGGGAATCTGCATGG-3′ and 5′-ATGTACACGATGTCTTTGGCAGATA-3′; NOS2, 5′-CAAGCACATTTGGGAATGGAGA-3′ and 5′- CAGAACTGAGGGTACATGCTGGAG-3′; GAPDH, 5ˊ- GAGCCAAACGGGTCATCATCT -3ˊand 5ˊ-GAGGGGCC ATCCACAGTCTT -3ˊ.

Briefly, the total RNA from lung tissues and macrophages was extracted in lysis buffer and purification of RNA was performed with RNeasy minicolumns following the manufacturer’s protocol (Fulengen Co., Ltd., Guangzhou). RNA was quantified using the NanoDrop ND-1000 spectrophotometer and amplified and biotin-labeled with Nugen’s Ovation System. The yield of total RNA per replicate varied from 0.6 μg to 2.0 μg. Fifty nanogram of the RNA was added in a SYBR qPCR master mix for real-time qPCR. Quantitative data for the Arg1 and NOS2 mRNA expression levels after normalizing to GAPDH were calculated as a percentage in reference to the expression level of the control sample.

### Statistics

Data were expressed mean ± standard deviation (SD) on experimental results. Statistical analysis was performed using Statistical Package for the Social Science (SPSS, version 19.0). Comparisons of individual groups were performed by one-way analysis of variance (ANOVA). Student’s paired t-test was used to compare measurements from groups. A *P* value of < 0.05 was considered significant.

## Results

### Characterization of allergen-induced airway response

**Fig. 1:**
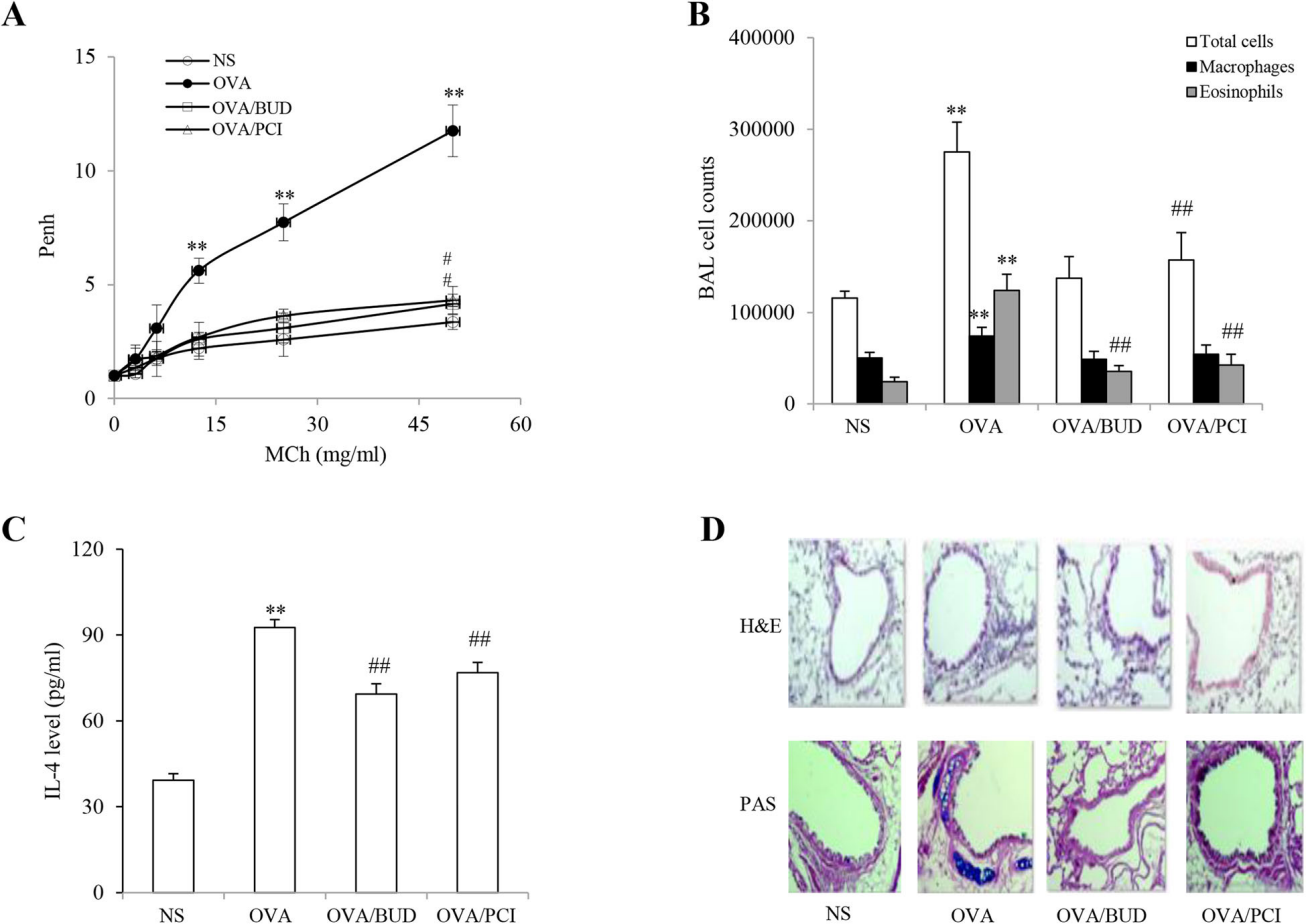
Mouse model of allergic asthma. Airway responsiveness (**a**), cell counts (**b**), IL-4 level (**c**) and morphological observation of lung tissues stained with H&E and PAS (**d**) were completed using different techniques. The results were expressed as Mean ± SD (*n* = 6). **: a *p*-value of < 0.01 vs NS, OVA/BUD and OVA/PCI. #, ##: a *p*-value of < 0.05 or 0.01 vs the control

AHR and airway allergic inflammation were measured in mice exposed to either NS or OVA in presence and absence of BUD or PCI-34051, respectively. The results are shown in Fig. 1. Repetitive measurements of Penh represented a gradual increase with increased concentrations of MCh in the mice. The ranking for the resistance measured from concentration-response curves was shown in such an order of OVA > PCI-34051 = BUD > NS in the airflow changes form the investigated animals. In contrast, the average value of Penn from OVA-challenged mice was a three-fold higher than the control mice at the highest dose of MCh (Fig. 1a). Although treatment with BUD and PIC resulted in obvious decreases in airway resistance in the animals exposed to OVA, the values were still higher than the control. In statistical analysis, there were significant differences in the values measured at the dosage levels (12.5, 25 and 50 mg/ml) of MCh inhalation between OVA group and other groups (all *P* < 0.01, *n* = 6). Additionally, there were differences seen in the Penh values at the maximum dose of MCh challenge between NS-treated mice and BUD- or PCI-treated ones (*P* < 0.05, *n* = 6).

The number of cells and cellular composition in the BALF sample were examined in each group. A large amount of the BALF cells in OVA-exposed mice was observed with increases of macrophages and eosinophils as compared to the control animals (Fig. 1b). All of the cell counts in OVA group showed a two-fold increase over other groups. Treatment with BUD and PCI obviously reduced the numbers of these cells in the samples from the challenged mice. In contrast, there were significantly statistical differences in the cell counts between OVA group and other groups (all *P* < 0.01, *n* = 6). Additionally, there were differences in the counts of total cells and eosinophils between NS group and either OVA/BUD or OVA/PCI group (*P* < 0.01, *n* = 6).

Serum IL-4 level (pg/ml) was determined in mice and the results displayed a high level of IL-4 in OVA-challenged mice (Fig. 1c). In contrast, the IL-4 level in the OVA-treated mice reached to 2.4, 1.3 and 1.2 folds over the NS-, BUD- and PCI-treated animals, respectively. There were statistically significant differences in the changes in the cytokine level between OVA group vs NS, OVA/BUD or OVA/PCI (*P* < 0.01, *n* = 6). Additionally, there were significant differences between NS group and either OVA/BUD or OVA/PCI group (*P* < 0.01, *n* = 6).

In histopathological examination, representative images of lung sections showed more severe infiltration of peribronchial inflammatory cells and a large amount of mucus secretion in the OVA-exposed lungs than the NS-treated lungs. Treatment with BUD and PCI-34051 resulted in significant reduction in the cell infiltration and mucus accumulation in the challenged lungs (Fig. 1d).

### HDAC8 and Galectin-3 expression in lungs and RAW264.7 cells

**Fig. 2:**
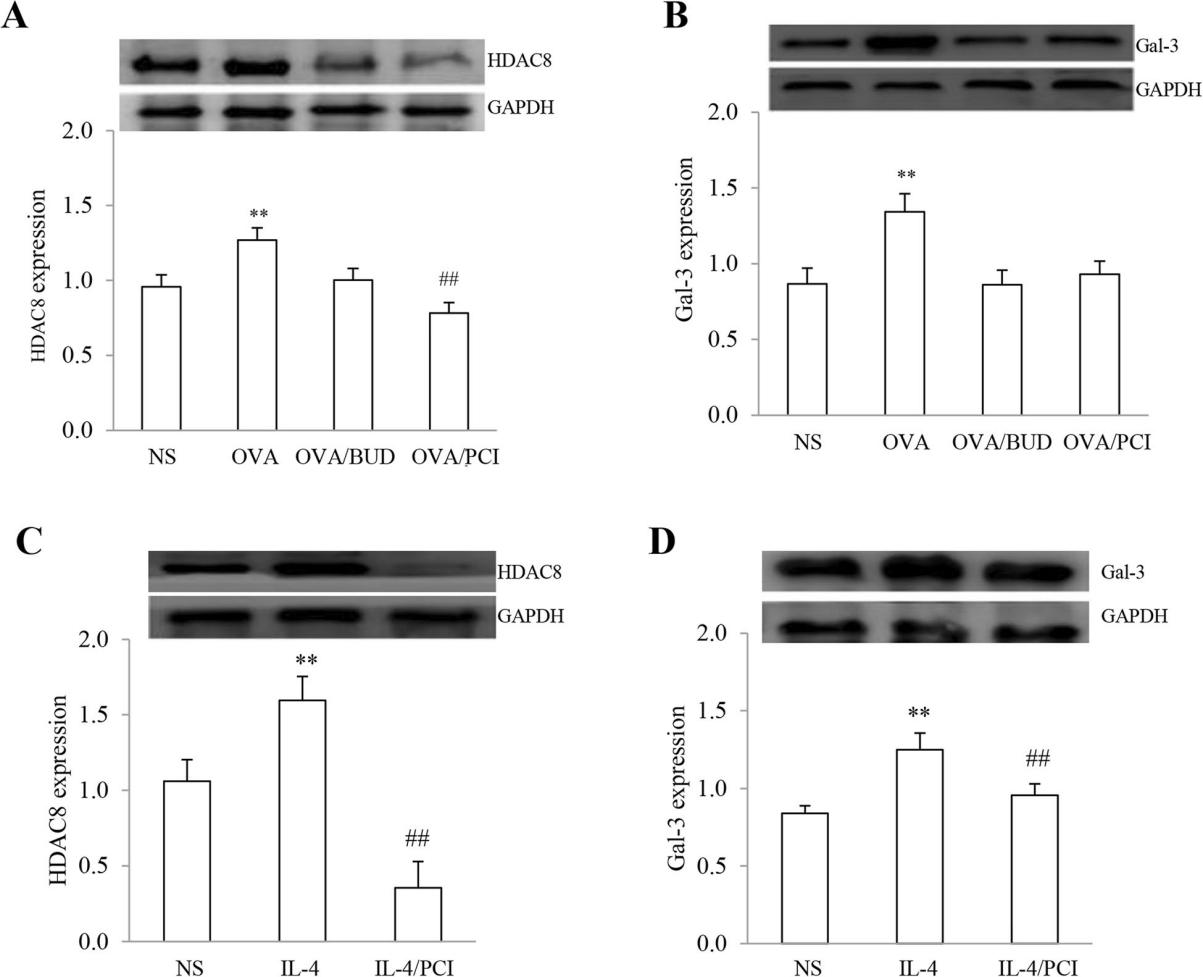
Expression of HDAC8 and Cal-3 in lung tissues and macrophages. HDAC8 and Cal-3 expression in lung specimens (**a**, **b**) and macrophages (**c**, **d**) were quantified using ImageJ and the value for the relative band intensity was calculated by each target protein band/GAPDH band. The results were expressed as Mean ± SD. **: *P* < 0.01 vs NS, OVA/BUD and OVA/PCI in the lung sample and vs NS or IL-4/PCI in the cells. (*n* = 3). ##: *P* < 0.01 vs NS (*n* = 3)

The expression levels of HDAC8 and Gal-3 in lung tissues and macrophages were examined by western blot procedures. The results are shown in Fig. 2. HDAC8 and Gal-3 expression in OVA-exposed lungs were higher than other groups. Treatment with BUD and PCI significantly reduced the protein expressions in the lungs (Fig. 2a, b).

Both proteins were also over-expressed in macrophages treated IL-4 as compared to the NS-treated cells (Fig. 2c, d). Treatment with PCI resulted in significantly reducing the expression levels of both proteins in the IL-4-treated cells. In statistical analysis, there were significant differences in the protein expressions in lung specimens between OVA group vs NS, OVA/BUD or OVA/PCI (all *P* < 0.01, *n* = 3) and NS group vs OVA/PCI group (*P* < 0.01). Furthermore, there were statistical differences in the protein expressions in the cells between IL-4 group vs NS or IL-4/PCI group (*P* < 0.01, *n* = 3) and NS group vs IL-4/PCI group (*P* < 0.01).

### Identification of macrophage phenotypes

**Fig. 3:**
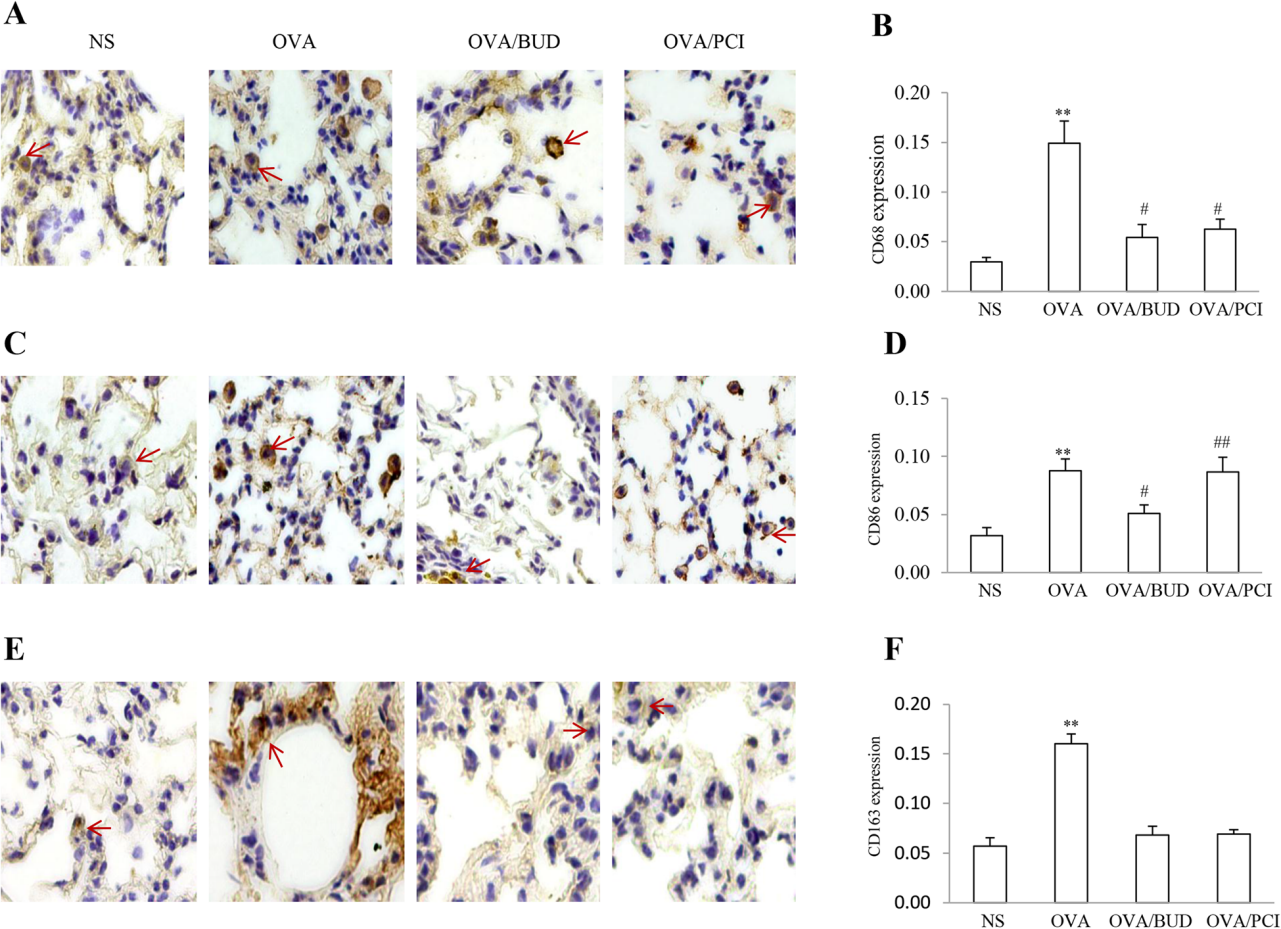
Macrophage polarization states. Distribution and proportion of CD68^+^ (**a**), CD86^+^ (**c**) and CD163^+^ (**e**) macrophages (brown) were identified using IHC procedures. Intensity for the color response to antibodies was visible in lung tissues from all groups. Population proportions for the stained cells as shown by red arrows were calculated as a fold change of the control. (**b**, **d**, **f**). The results were expressed as Mean ± SD (*n* = 3). **: a *p*-value of < 0.01 vs NS, OVA/BUD and OVA/PCI except the number of CD86^+^ cells in OVA/PCI group. #: *P* < 0.05 vs NS and ##; *P* < 0.01 vs either NS or OVA/BUD group

CD molecules can act in numerous ways, often acting as cell surface markers used to identify macrophage populations. Polarized macrophage subsets in lung specimens were recognized by appearance as a typical color response according to the features of antibodies and the results are shown in Fig. 3. CD68^+^, CD86^+^ and CD163^+^ cells presented in a brown color under a light microscope. In analysis of the expression levels of the cell surface markers, the quantity of the cells and stain concentration of these three markers significantly increased in OVA-exposed lungs by visual discrimination (Fig. 3a, c, e). The color responses to antibodies became lighter in the samples from NS-, BUD- and PCI-treated mice without a proportional change. In quantitative analysis of color intensity, population proportions of CD68^+^, CD86^+^ and CD163^+^ cells in lungs from OVA group was larger than these in other groups except distribution proportion of the CD86^+^ cells in the OVA/PCI group (Fig. 3b, d, f). Treatment with BUD and PCI predominantly reduced the number of CD68^+^ and CD163^+^ cells in the challenged lungs. However, proportion of CD86^+^ cells in the PCI-treated mice was unchanged with the change as same as the OVA-treated mice. In contrast, there were statistical differences in the proportion distribution of CD68^+^ and CD163^+^ macrophages between OVA group and NS, OVA/BUD or OVA/PCI group (all *P* < 0.01, *n* = 3). Moreover, it was worth to note that there was no difference in the fraction of the CD86^+^ cells between OVA group and OVA/PCI group.

### Arg1 and NOS2 protein expression in lung specimens and macrophages

**Fig. 4:**
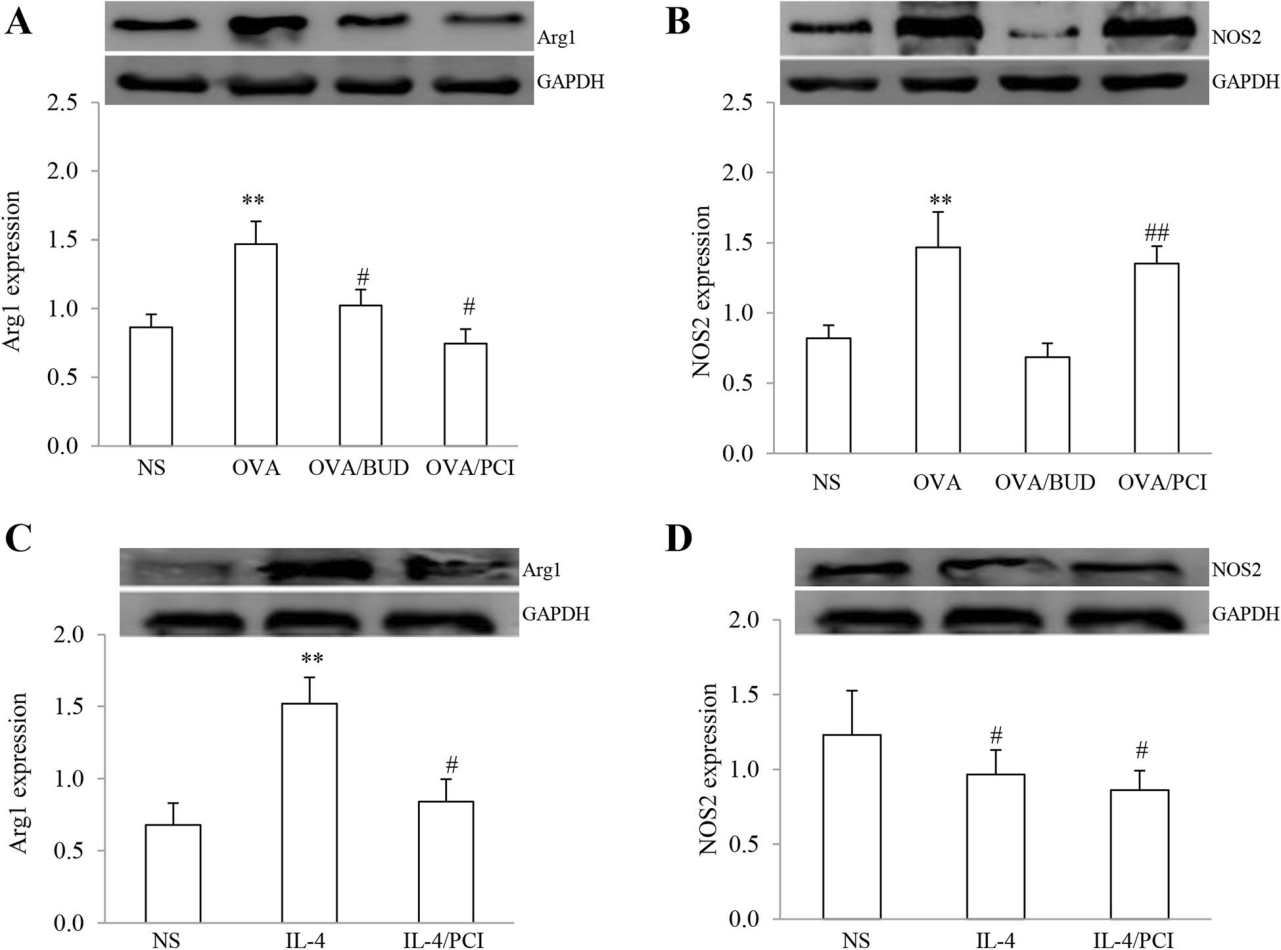
Arg1 and NOS2 protein expression in lungs and macrophages. Arg1 and NOS2 proteins in lung tissues (**a**, **b**) and macrophages (**c**, **d**) were present on images and quantitation of both proteins was executed by calculating the value from the relative band intensity of each target protein/GAPDH protein. The results were expressed as Mean ± SD (*n* = 3). **: *P* < 0.01 vs NS, OVA/BUD and OVA/PCI except NOS2 in PCI-treated lungs or vs NS and IL-4/PCI in the cells. #, ##: *P* < 0.05 or 0.01 vs NS

Arg1 and NOS2 proteins in lung tissues and macrophages were quantitated by western blot analysis and the results are shown in Fig. 4. Increases in both protein expression levels were visible in representative gel images in OVA-exposed lung tissues and the cells treated with IL-4 as compared to other treatments except the NOS2 expression in the image from PCI-treated mice. These findings were also evidenced by quantitative analysis of band intensity. The average value for intensity of Arg1 and NOS2 protein expression was shown as 0.86 + 0.10 and 0.82 + 0.09 in NS, 1.47 + 0.16 and 1.47 + 0.25 in OVA, 1.02 + 0.12 and 0.68 + 0.10 in OVA/BUD and 0.74 + 0.11 and 1.35 + 0.12 in OVA/PCI, respectively. The expression levels of both proteins in lungs from OVA group were higher than from NS group (Fig. 4a, b). In contrast to these findings from the tissues, the expression level of Arg1 in the IL-4-treated macrophages was significantly increased as compared to NS- and PCI-treated cells (Fig. 4c). In contrast, NOS2 in the cells treated with IL-4 was expressed at a low level (Fig. 4d). The average value for both protein expression levels in macrophages was displayed as 0.68 + 0.15 and 1.23 + 0.3 in NS, 1.52 + 0.18 and 0.97 + 0.16 in IL-4 and 0.84 + 0.15 and 0.86 + 0.13 in IL-4/PCI. Treatment with BUD suppressed these two protein expressions in the challenged lungs (both *P* < 0.01, *n* = 3). However, PCI intervention merely reduced Arg1 but not NOS2 expression in the lungs and the IL-4-treated cells (*P* < 0.05 or 0.01, *n* = 3).

### Arg1mRNA and NOS2 mRNA expression in lung specimens and macrophages

**Fig. 5:**
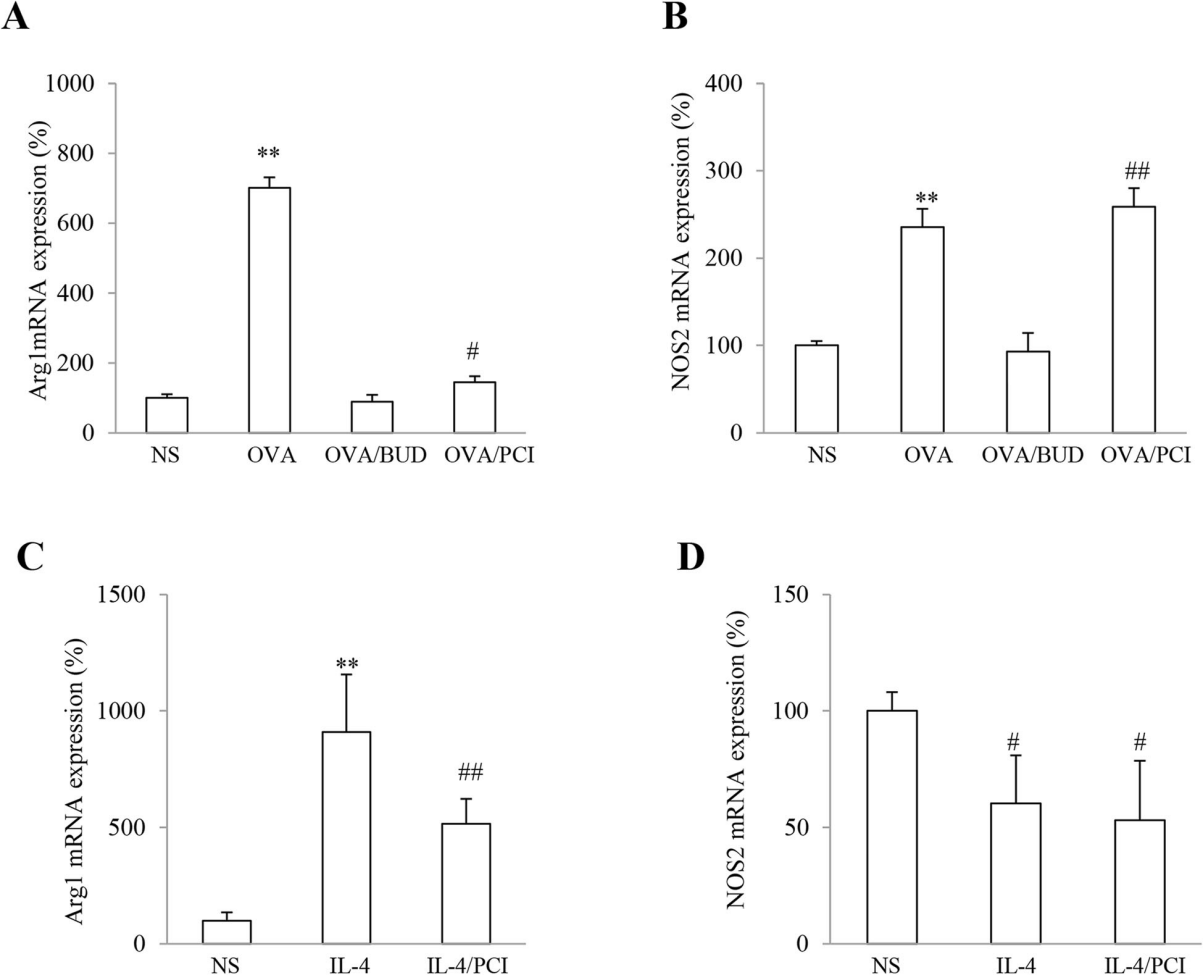
Arg1 and NOS2 mRNA expression in lungs and macrophages. The Arg1 and NOS2 mRNA levels in lung tissues (**a**, **b**) and macrophages (**c**, **d**) were expressed as a percentage (%) of the mRNA level of the control. The results were expressed as Mean ± SD (*n* = 3). **: *P* < 0.01 vs NS, OVA/BUD and OVA/PCI except the NOS2 mRNA level in PCI-treated lungs or vs NS and IL-4/PCI. #, ##: *P* < 0.05 or 0.01 vs either NS or BUD group (*n* = 3)

Frequency of gene expression in lung tissues was examined by transcriptional profiling and transferred to a percentage of the mRNA expression level as compared to the mRNA level in the control sample. The results are shown in Fig. 5. Average values (%) for Arg1 and NOS2 mRNA levels in lung tissues were displayed as 100.00 + 10.01 and 100.00 + 4.99 in NS, 700.95 + 30.33 and 235.29 + 21.19 in OVA, 88.92 + 19.25 and 93.02 + 23.09 in OVA/BUD and 144.57 + 17.07 and 258.86 + 25.53 in OVA/PCI, respectively. In general, both gene expression levels were significantly increased in OVA group as compared to NS group (Fig. 5a, b). Treatment with BUD resulted in significant decreases in the Arg1 and NOS2 mRNA levels (both *P* < 0.01, *n* = 3). However, PCI intervention only reduced the Arg1 mRNA but not NOS2 level (*P* < 0.01, *n* = 3).

In terms of macrophages, Arg1 gene expression was enhanced with a decrease in NOS2 expression in the cells treated with IL-4 as compared to other treatments (Fig. 5c, d). The average values for Arg1 and NOS2 mRNA levels were shown as 100.00 + 34.75 and 100.00 + 8.0 in NS, 904.79 + 247.97 and 60.27 + 20.68 in IL-4 and 506.70 + 96.86 and 53.10 + 25.52 in IL-4/PCI. There were statistically significant differences observed in the Arg1 and NOS2 mRNA expression levels between NS vs IL-4 and NS vs IL-4/PCI (*P* < 0.05 or 0.01, *n* = 3).

### Detection of two distinct antigens in lung specimens and macrophages

**Fig. 6:**
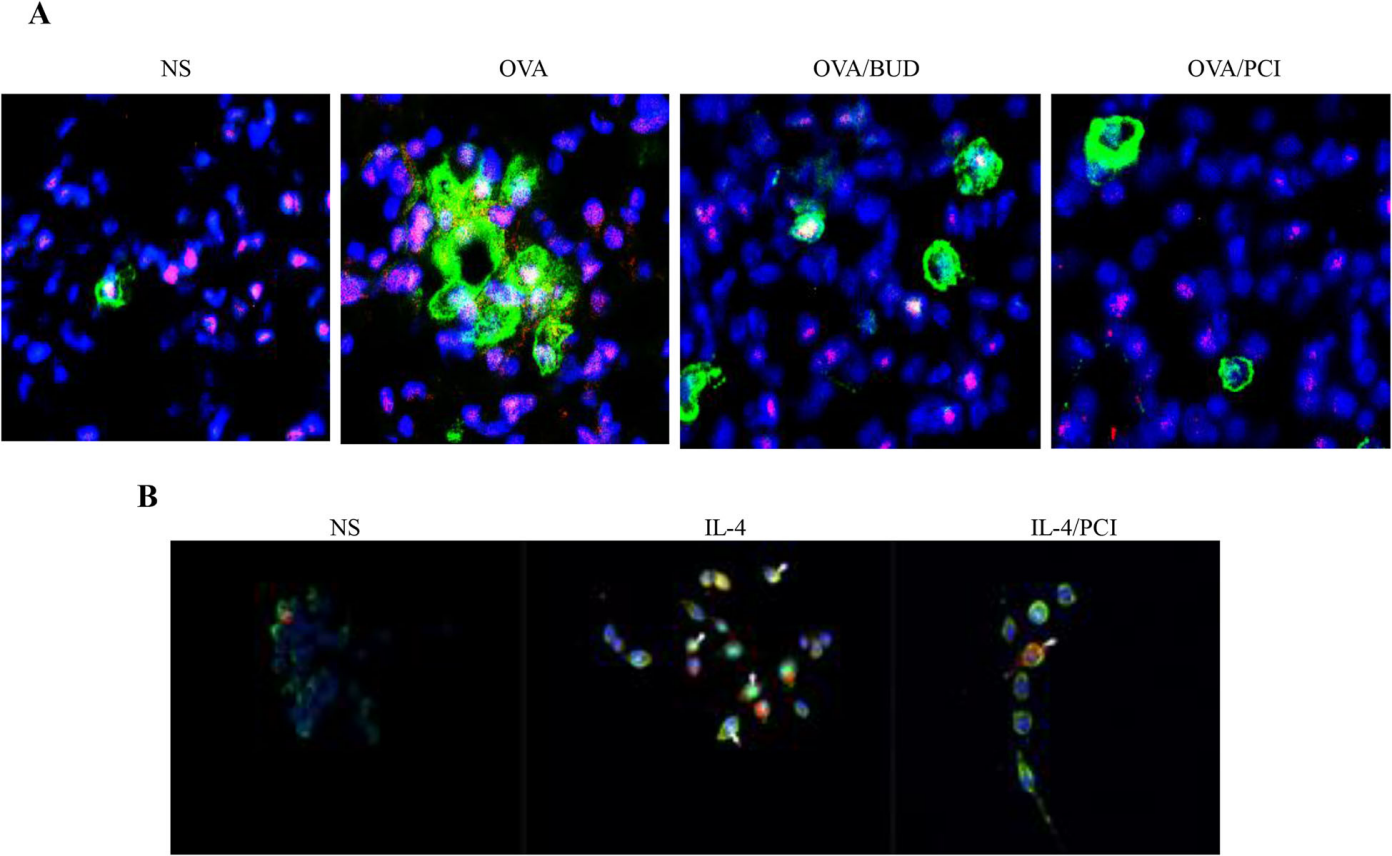
Double immunofluorescent staining of HDAC8 and Cal-3 expression. Expressions of HDAC8 (bright orange) and Cal-3 (green) in lung specimens (**a**) and macrophages (**b**) were identified according to the image results. An increase in the HDAC8 expression level was synchronously associated with the same change of Cal-3 expression in the OVA-treated lungs and IL-4-treated cells. Treatment with BUD and PCI resulted in visualising parallel reduction of the expression levels of both proteins in the investigated samples

To investigate HDAC8 and Cal-3 interaction, protein expression states in lung tissues and macrophages were examined by double staining IF and the results are shown in Fig. 6. The double protocol successfully stained HDAC8 (bright orange) and Cal-3 (green) on the investigated samples, revealing each specific protein presented in the samples in the light of the visible identification marks. The green-tagged protein (Gal-3) was found both in cytoplasm and nucleus of lung epithelial cells and macrophages, whereas the bright orange-tagged protein (HDAC8) was only expressed in the nucleus. In this assays, the color tags were widespread in the lung specimens and the cells, whereas both colors as target labels obviously gathered in OVA-exposed lungs and IL-4-treated cells. In image analysis, HDAC8 expression synchronously associated with the change of the Gal-3 state in vision. Treatment with BUD and PCI significantly reduced the expressions of the labeling proteins in the lungs and cells at the same time.

### Detection of interacting protein

**Fig. 7:**
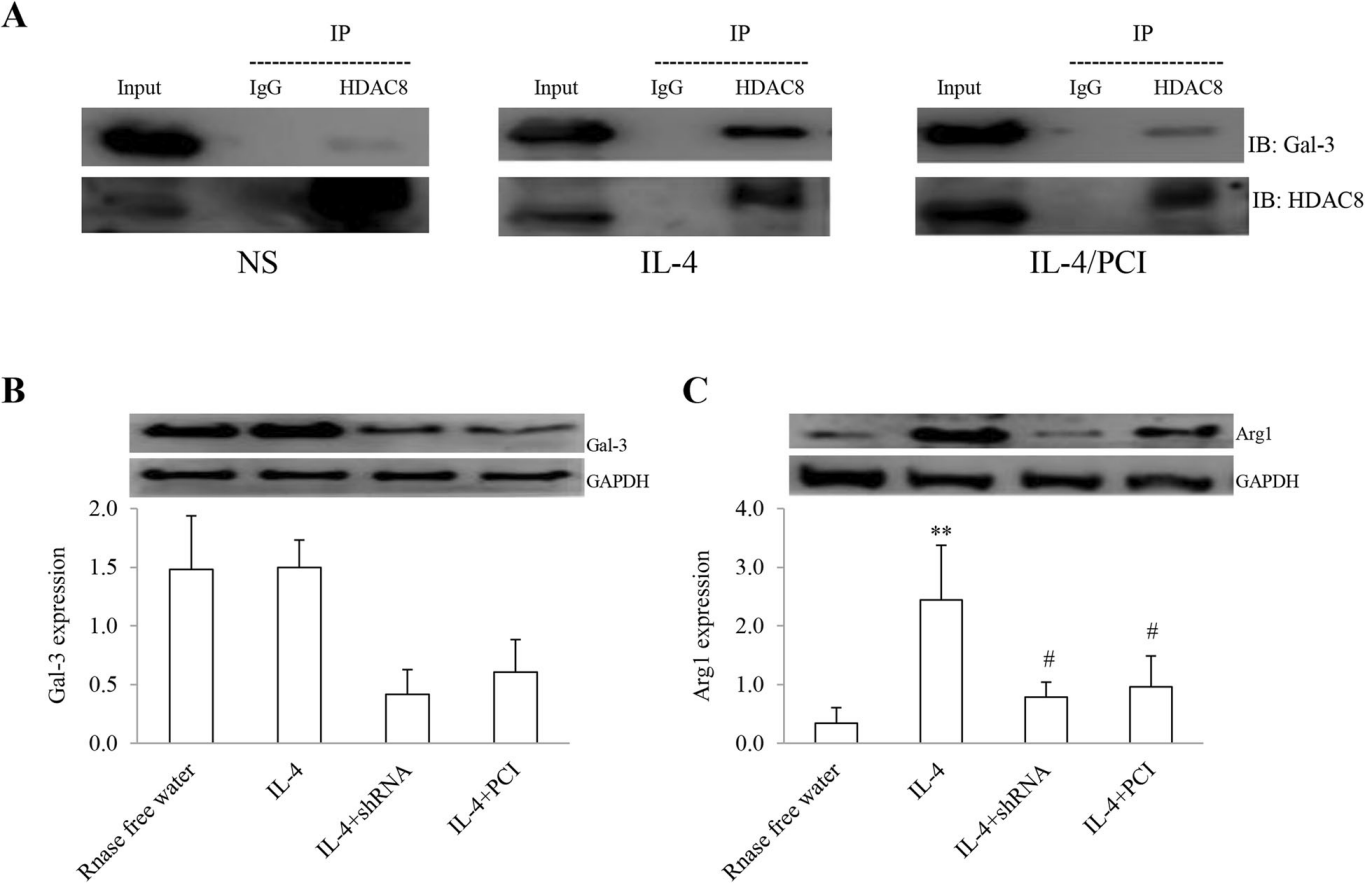
Identification of HDAC8 and Gal-3 interaction. HDAC8 and Gal-3 (**a**) in protein complex from cell lysate of macrophages was immunoprecipitated (IP) with anti-HDAC8 and anti-IgG, identified by immunoblotting (IB). Input (lysates) was loaded with same amount of protein in sample. An isotype IgG was a negative control. The expression levels of Gal-3 (**b**) and Arg1 (**c**) in macrophages were determined by shRNA and PCI. The results were expressed as Mean ± SD (*n* = 3). **: *P* < 0.01 vs RNase free H_2_O, IL-4/shRNA and IL-4/PCI. #: *P* < 0.05 vs the control (*n* = 3)

Co-IP and gene silencing are typically used to analyze protein-protein interactions and the state of gene expression in macrophages. The results are shown in Fig. 7. Interacting proteins were identified by using target protein-specific antibodies (Fig. 7a). In contrast to the NS-treated macrophages, the two protein bands for HDAC8 and Gal-3 expressions were clearly detected in the IL-4-treated cells, whereas treatment with PCI resulted in synchronously decreasing these two proteins expression in the cells.

To observe the subsequent effects of the both protein interaction, the Arg1 expression level was determined by using the shRNA technology. In the preliminary experiment, Gal-3 was knocked down by 73% or 72% in its expression level as compared to the control (Fig. 7b). In further experiments, Arg1 expression was significantly increased in the cells treated with IL-4 alone as compared to the cells treated with RNase H_2_O (Fig. 7c). However, the expression level was significantly reduced in the cells treated with shRNA1 or PCI. There was statistically significant difference in the protein expression between IL-4 group vs RNase free H_2_O, IL-4/shRNA and IL-4/PCI (*P* < 0.01, *n* = 3) and the control vs IL-4/shRNA or IL-4/PCI (*P* < 0.05).

## Discussion

To gain an insight into the roles of HDAC8 in the pathologic process of allergic asthma, a mouse model of the allergic response to inhaled allergen was established. The OVA-exposed mice reproduced many key features of clinical asthma including AHR, large amount of eosinophils recruited to BALF and excess mucus accumulated in the inflamed airways [25, 26]. Additionally, it was worth noting that there were a large amount of alveolar macrophages (AMs) and an elevated IL-4 level detected in the BALF samples. These features of this model were relieved by the treatment with BUD or PCI, indicating that the HDAC8 inhibitor served as reliable tool with utility in understanding the underlying pathologic changes of the asthma. Mouse models of the allergic response to inhaled OVA have been widely used to elucidate the mechanisms underlying the inflammatory responses in asthma [27, 28]. Furthermore, IL-4 induces AM activation [29, 30]. The model therefore helps identify AM phenotypic and functional transformation in airway inflammation and provides a pathologic link between AMs and IL-4.

HDAC8 and Gal-3 proteins in OVA-challenged lungs and IL-4-treated macrophages were overexpressed as compared to other treatments, revealing apparent association concerning functional activity of the two proteins in the investigated samples. The finding led us to speculate that mediating interaction between HDAC8 and Gal-3 participates in the inflammatory settings. Treatment with PCI significantly suppressed these protein expressions in the tissues and cells, further suggesting simultaneous but not sequential interactions existed between the two proteins. Though HDAC8 and Gal-3 have been reported as factors involved in pathological process of allergic asthma [15, 31–33], a combined pattern of HDAC8 interacting with Gal-3 is still unclear in asthma.

Given a large amount of AMs recruited to lungs in this model, functional phenotypes of AM polarization required to be categorized and identified according to the markers of CD68 (M0, macrophage), CD86 (M1, classically activated macrophage) and CD163 (M2, alternatively activated macrophage) [34–36]. The results manifested that population proportions for all three subsets of AMs significantly enhanced in OVA-exposed lungs, demonstrating that AMs implicate in development of ongoing airway inflammation and serve as a line of defense against foreign invaders to the lung tissue [23, 37]. Recruited AMs possess diverse functions relating to their activation states, which have been termed as M1 or M2 macrophages derived from M0 AMs [38]. Though the origin of AMs is not fully elucidated, evidence indicates that these cells are originated from either differentiation of blood monocytes or proliferation of resident AMs [39]. M1 macrophages are known to drive inflammation in response to intracellular pathogens and express NOS2 [40, 41], while M2 macrophages involve in anti-inflammatory responses and up-regulate Agr1 expression [41, 42]. PCI intervention reduced the expressions of CD68^+^ and CD163^+^ cells but not CD86^+^ cells, indicating that resident macrophages as initial responders to antigenic stimuli participated in pathogenesis of allergic asthma and a turnover rate of M2 AMs was down-regulated in the presence of the HDAC8 inhibitor. Based on the above findings, it led us to conclude that M2 polarization was a prominent process of ongoing airway inflammation and the dynamic changes in an activation state of the cells referred to HDAC8 and Gal-3 expression. M2 macrophages are abundantly present in lungs of asthma models and asthmatic patients [43, 44], whereas excessive M2 macrophage activity leads to asthma [45]. Additionally, M2 macrophages induce FIZZ1 expression which increases airway smooth muscle force generation [46, 47]. Even though PCI reduced the numbers of CD163-expressing cells, it was still lacking in identification at molecular levels.

Arg1 and NOS2 expression were determined at their protein and mRNA levels. The results showed that Arg1 protein and mRNA levels were increased in both OVA-exposed lungs and IL-4 treated macrophages, indicating phenotype and function of the cells in the investigated samples. Since Arg1 is a prototypic marker for M2 activation and IL-4 may induce macrophage differentiation to M2 phenotypes in asthma [37, 48], the consistency regarding these findings between lung tissues and cells supported the consideration that M2 polarization could be of particular importance in the Th2 pathology asthma since IL-4 is a key cytokine in the development of allergic inflammation [44, 49]. Notably, NOS2 expression at the protein and mRNA levels was increased in OVA-exposed lungs but not in IL-4-trerated cells. The difference between the tissue and the cells was caused probably due to the fact that NOS2 existed in airway epithelium in the lung sample [50]. Treatment with PCI suppressed Arg1 but not NOS2 expression, suggesting that HDAC8 and Gal-3 conduced to polarization of macrophages toward the M2 phenotype. Furthermore, the molecular marker assay for NOS2 at the cell level was more reliable than at the tissue level in identifying macrophage phenotypes.

Double immunofluorescence protocol was employed to explore HDAC8-Gal-3 interaction within lung tissues and macrophages. Our results indicated that color tags for these two proteins were widespread in the investigated samples. Gal-3 was found both in cytoplasm and nucleus of lung epithelial cells and macrophages, whereas HDAC8 was only expressed in the nucleus, demonstrating that HDAC8 is a nuclear-localized protein. In contrast to the visibility, the color responses to antibodies were much stronger in OVA-exposed lungs and IL-4-treated cells than in the control samples. More surprisingly, treatment with BUD or PCI resulted in visually parallel reduction of color intensity of HDAC8 and Gal-3 in the lungs and cells, indicating synchronous interference occurred on the two targets in the same samples. Double immunofluoresence staining has been applied for examining distribution of two different antigens in the same tissue which retains excellent morphology [51, 52]. Moreover, the color labels for two primary antibodies can be detected simultaneously using an antibody cocktail by probing antigens [53]. Our results not only provide insight into the localization of independent antigens within the tissue and cells but also suggest that HDAC8 and Gal-3 as convergent targets were co-interfered in the antigen-induced lung disease.

The components in protein complexes were isolated and identified using Co-IP technique followed by western blotting analysis. The results revealed that HDAC8 and Gal-3 existed in the complex and both protein expressions were synchronously up-regulated in IL-4 treated cells and down-regulated in the cells treated with PCI. It has been reported that Gal-3 contains a single carbohydrate recognition domain with an extended N-terminus which can interact with both carbohydrates and intracellular proteins [54]. The molecular structure of the protein could explain the critical role of these regions in protein-protein interactions. Given these findings, it leads us to conclude that HDAC8 interacts with Gal-3 in the process of IL4-induced M2 macrophage polarization. In other words, the changes in functional activity of the IL-4-treated macrophages require interaction of HDAC8 with Gal-3 [40]. Since biological responses related to airway inflammation present active regulation of the cellular proteome [55], we examined Agr1 expression after knocking down Gal-3 and use of PCI. In contrast to Arg1 overexpressed in the IL-4-derived macrophages, Gal-3 knockdown or HDAC8 inhibitor significantly reduced the enzyme expression, suggesting that the nuclear-localized protein HDAC8 is a prerequisite for the suppressive function in the proteome expression pattern because protein interaction requires the physical contacts of molecules and shares a common subcellular location [56, 57]. Though it is unclear how HDAC8 binds to Gal-3 in precise mechanism, our results provide evidence that HDAC8-Gal-3 interaction would occur in the nucleus. It has been reported that polyamines produced by macrophage arginase-1 may attract and activate mast cells, thus promoting airway inflammation [58]. Since PCI intervention synchronously reduced HDAC8 and Gal-3 expression, it’s reasonable to speculate that therapies directed at disruption of the protein interaction patterns may prove to be of benefit in AHR and airway inflammation.

## Conclusions

The HDAC8 inhibitor attenuates AHR and airway inflammation in the animal model of allergic asthma through suppressing HDAC8-Gal-3 interaction and reducing M2 macrophage polarization.

## Data Availability

All data generated or analyzed during this study are included in this published article.

## Abbreviations

AHR: Airway hyperresponsiveness
BALF: Bronchoalveolar lavage fluid
BUD: Budesonide
Co-IP: Co-immunoprecipitation assay
DMEM: Dulbecco’s Modified Eagle’sMedium
H&E: Hematoxylin and eosin
HDAC: Histone deacetylase
IF: Immunofluorescence
IHC: Immunocytochemistry
NS: Normal saline
OVA: Ovalbumin
PAS: Periodic Acid-Schiff
